# Collective Motion in a Network of Self-Propelled Agent Systems

**DOI:** 10.1371/journal.pone.0144153

**Published:** 2015-12-07

**Authors:** Hao Peng, Dandan Zhao, Xueming Liu, Jianxi Gao

**Affiliations:** 1 Department of Computer Science and Engineering, Zhejiang Normal University, Jinhua 321004, Zhejiang, P. R. China; 2 Key Laboratory of Image Information Processing and Intelligent Control, School of Automation, Huazhong University of Science and Technology, Wuhan 430074, Hubei, China; 3 Center for Polymer Studies and Department of Physics, Boston University, Boston, Massachusetts 02215, United States of America; 4 Center for Complex Network Research and Department of Physics, Northeastern University, Boston, Massachusetts 02115, United States of America; Lanzhou university of Technology, CHINA

## Abstract

Collective motions of animals that move towards the same direction is a conspicuous feature in nature. Such groups of animals are called a self-propelled agent (SPA) systems. Many studies have been focused on the synchronization of isolated SPA systems. In real scenarios, different SPA systems are coupled with each other forming a network of SPA systems. For example, a flock of birds and a school of fish show predator-prey relationships and different groups of birds may compete for food. In this work, we propose a general framework to study the collective motion of coupled self-propelled agent systems. Especially, we study how three different connections between SPA systems: symbiosis, predator-prey, and competition influence the synchronization of the network of SPA systems. We find that a network of SPA systems coupled with symbiosis relationship arrive at a complete synchronization as all its subsystems showing a complete synchronization; a network of SPA systems coupled by predator-prey relationship can not reach a complete synchronization and its subsystems converges to different synchronized directions; and the competitive relationship between SPA systems could increase the synchronization of each SPA systems, while the network of SPA systems coupled by competitive relationships shows an optimal synchronization for small coupling strength, indicating that small competition promotes the synchronization of the entire system.

## Introduction

Groups of animals sometimes display fascinating collective motions [1–6] in which animals move in the same direction, such as schools of fish can move in a rather orderly fashion or change direction amazingly abruptly [2], and flocks of birds can fly as a uniformly moving group. Moreover, collective motion are common both in living and non-living worlds, ranging from biology [7], ecology [8], climate [9], society to technology [10, 11] and even art [12], the studies of which can help us understand the nature world and improving infrastructure systems in the man-made world. A simple model proposed by Vicsek et al. [13], unveiling the collective motion and phase transition of self-propelled agent systems (SPA), can be potentially applied to man-made systems, such as distributed sensor networks [14], unmanned aerial vehicles [15], underwater vehicles [16], altitude alignment of satellite clusters [17], and many more. Underlying the behavior of collective motions, synchronization process is the cause of such fascinating phenomenon. Various models have been proposed to mimic synchronization processes and many strategies has presented to improve the properties of synchronization [18, 19]. Most of these studies are focus on isolated networks.

Increasing evidence shows that one system may interact or couple with other systems, such as: different social networks (e.g., Facebook, Twitter) are interconnected with each other because the nodes in different networks share the same actors [20]; transportation networks (e.g., buses, airplanes) are coupled with each other since the nodes in each network are in the same geographic locations [21]; the infrastructure systems (e.g. communication networks and power grid) are interdependent because the nodes in one network support the nodes in another network [22, 23]. Indeed, some realistic systems such as neuronal system could also be self-propelled or self-adjusting due to the autapse connection to neuron, that the collective behaviors of neurons could be regulated by autapse driving when continuous pulse or traveling wave is induced [24, 25]. All these examples unveil that real systems usually interact with each other, leading to the emerging new field in network science, interdependent networks, interconnected networks, a network of networks, multi-layered networks, multiplex networks and many more [26–32]. In real scenarios, different self-propelled agent systems also coupled with each other and form a network of SPA systems. For example, unmanned aerial vehicles may work with underwater vehicles to achieve some tasks, exhibiting a symbiotic relationship between these SPA systems; a flock of birds might have predator-prey relationships with a school of fish; and schools of fish may compete with each other for sharing the same food. Such coupling relations between different SPA systems could influence the synchronization of these systems. However, no such model exists for showing how the interdependence between different SPA systems influence the synchronization of a network of SPA systems. Studies of synchronization of network of SPA systems enable us to design high efficient systems of different coupled unmanned vehicles or robots.

In this work, we propose a model to show the collective motion in a network of SPA systems, generalizing the Vicsek model [13] (VM). In the model, we introduce a coupling strength *β* ∈ [0, 1] denoting the ratio of a node’s the radius in its coupled system to that in its current subsystem. Furthermore, we construct three networks of SPA systems coupled by three types of interaction: symbiosis, predator-prey, and competition. Besides, we find that (1) there exits an optimal coupling strength in the coupled systems with symbiotic and predator-prey relationship to achieve optimal synchronization for each subsystem and the entire system; (2) furthermore, increasing the radius and absolute velocity and decreasing of system size could increase the optimal synchronization of every subsystem and the entire systems; (3) in the systems coupled by competitive relationships, increasing the coupling strength could increase the synchronization of each subsystem but decrease the synchronization of the entire system.

## Model

**Fig 1:**
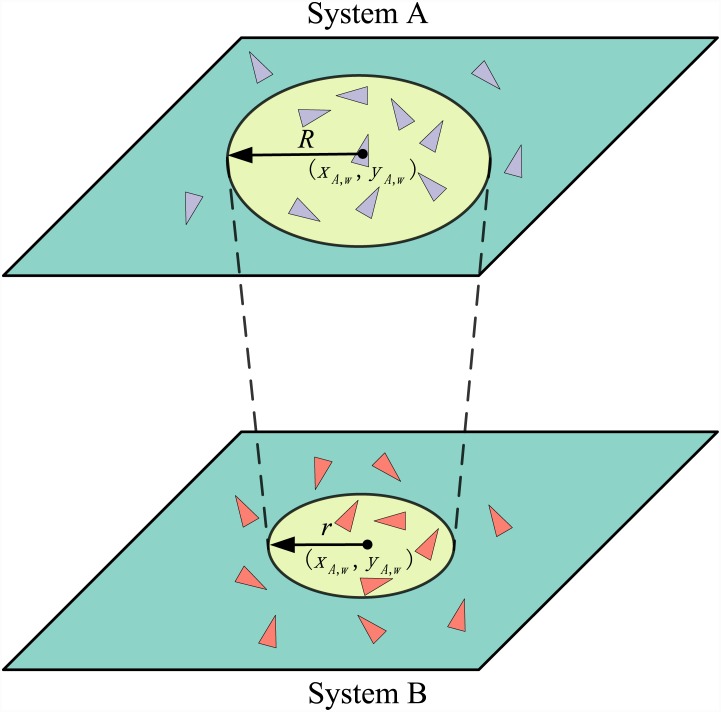
In system A, the position of node *w* is (*x*_*A*,*w*_,*y*_*A*,*w*_), and it has 7 neighboring agents within a circle with a radius being *R* [Eq (3)], whose position projecting on system B is also (*x*_*A*,*w*_,*y*_*A*,*w*_), and it has 4 neighboring agents in system B [Eq (4)]. Accordingly, we could identify the neighborhoods of all nodes in both systems.

Our model contains *N* coupled subsystems of self-propelled agents with size *n*_*k*_ in subsystem *k*, where the agents in each subsystem move in a *L* × *L* square with a same constant speed towards different directions. Initially, the agents are randomly distributed on the *L* × *L* square plane, and their directions are also uniformly randomly distributed in the interval (0, 2*π*). At each time step, the direction of each agent is determined by the average directions of all the agent within a circle centered at the given agent with a radius *R* (Fig 1 System A). At each step *t*, the position of a specific agent is updated according to

$$x_{w}\left(t+1\right)=x_{w}\left(t\right)+v_{0}e^{i\theta_{w}\left(t\right)},$$ (1)

where *x*_*w*_(*t*) is the two dimensional vector of position of agent w at time *t*, *v*_0_ is the absolute speed of each agent and *θ*_*w*_(*t*) is the direction of agent *w* at time *t*. Then its direction is updated following

$$e^{i\theta_{w}\left(t+1\right)}=e^{i\Delta \theta_{w}\left(t\right)}\frac{\sum_{j\in \Gamma_{w}\left(t+1\right)}e^{i\theta_{j}\left(t\right)}}{\|\sum_{j\in \Gamma_{w}\left(t+1\right)}e^{i\theta_{j}\left(t\right)}\|},$$ (2)

where ‖⋅‖ is the standard norm [33] defined by ‖(*z*_1_, *z*_2_, …, *z*_*l*_)‖ = (|*z*_1_|^2^ + |*z*_2_|^2^ + … + |*z*_*l*_|^2^)^1/2^, △*θ*_*w*_ ∈ [−*η*, *η*] representing the white noise, $e^{i\theta_{w}\left(t\right)}$ is a unit directional vector, and Γ_*w*_(*t*+1) is the set of neighbors of agent *i* at time step *t*+1, defined as

$$\Gamma_{Aw}\left(t\right)=\left\{j|{\left(x_{A,w}\left(t\right)-x_{A,j}\left(t\right)\right)}^{2}+{\left(y_{A,w}\left(t\right)-y_{A,j}\left(t\right)\right)}^{2}\leq R^{2}\right\},$$ (3)

and

$$\Gamma_{Bw}\left(t\right)=\left\{j|{\left(x_{B,w}\left(t\right)-x_{B,j}\left(t\right)\right)}^{2}+{\left(y_{B,w}\left(t\right)-y_{B,j}\left(t\right)\right)}^{2}\leq R^{2}\right\}$$ (4)

for system A and B respectively. In order to measure the synchronization of the system, an order parameter is introduced as [13, 34]:

$$V_{\alpha k}=\frac{1}{n_{k}}\left\|\sum_{w=1}^{n}e^{i\theta_{w}\left(t\right)}\right\|,\;0\leq V_{\alpha k}\leq 1.$$ (5)

A larger value of *V*_*αk*_ indicates that the subsystem *k* shows a better synchronization, and when *V*_*αk*_ = 1 all the agent are moving towards the same direction. A system of two coupled SPA systems (A and B) is shown in Fig 1, where each SPA system forms a subsystem. An agent *A*_*w*_ in system A moves inside a *L* × *L* square and its position is (*x*_*A*,*w*_, *y*_*A*,*w*_). The neighborhood of node *A*_*w*_ in system B is defined as

$$\Omega_{AB,w}\left(t\right)=\left\{k|{\left(x_{A,w}\left(t\right)-x_{B,k}\left(t\right)\right)}^{2}+{\left(y_{A,w}\left(t\right)-y_{B,k}\left(t\right)\right)}^{2}\leq r^{2}\right\},$$ (6)

and the neighborhood of node *B*_*w*_ in system A is defined as

$$\Omega_{BA,w}\left(t\right)=\left\{k|{\left(x_{B,w}\left(t\right)-x_{A,k}\left(t\right)\right)}^{2}+{\left(y_{B,w}\left(t\right)-y_{A,k}\left(t\right)\right)}^{2}\leq r^{2}\right\},$$ (7)

where *r* = *β* × *R*. Especially, (i) when *β* = 0, the two SPA systems are separated from each other corresponding to the case of two isolated SPA systems; (ii) when *β* = 1, the entire system fully mixed with each other forming a new subsystem with the density being the sum of these two SPA subsystems. When *β* > 0, an agent *w* in system A updates its direction according to both the agents in Γ_*Aw*_ and *Ω*_*AB*,*w*_ together, and updates its location according to Eq (1). Furthermore, different impact of an agent from one system on an agent from another system exists according to different relationships between these two SPA systems, which will be discussed as follows.

### (1) Symbiotic relationship

A symbiotic relationship between two coupled systems benefit the agents of both systems [35]. The agents of one system have positive impact to the synchronization of the agents in another system, leading a result that an agent *w* in system A changes its direction according to the average values of directions of all the agents in both Γ_*Aw*_ and *Ω*_*AB*,*w*_ as

$$e^{i\theta_{A,w}\left(t+1\right)}=e^{i\Delta \theta_{A,w}\left(t\right)}\frac{\sum_{j\in \Gamma_{Aw}\left(t+1\right)}e^{i\theta_{A,j}\left(t\right)}+\sum_{j\in \Omega_{AB,w}\left(t+1\right)}e^{i\theta_{B,j}\left(t\right)}}{\|\sum_{j\in \Gamma_{Aw}\left(t+1\right)}e^{i\theta_{A,j}\left(t\right)}+\sum_{j\in \Omega_{AB,w}\left(t+1\right)}e^{i\theta_{B,j}\left(t\right)}\|}.$$ (8)

According to Eq (8), one can obtain the direction changes of agents in system B symmetrically. Note that this model can be potentially applied to the systems where both subsystems intend to achieve a global alignment, such as the system of unmanned aerial vehicles and underwater vehicles. Furthermore, this model is similar to the classic model of interdependent networks [21] where two nodes from different networks depending on each other because they support each other showing a symbiotic relationship.

### (2) Predator-prey relationship

In the model where SPA systems coupled by predator-prey relationship [36], without lose of generality, we assume that system A is composed by the predators, and system B is composed by the preys. Then each agent of system A updates its direction by the average directions of all its neighbors in both systems A and B, because predators intends to synchronize with the preys; and each agent in system B updates its direction using the average directions of all its neighbors in system B and average of all its neighbors’ opposite directions in system A, since preys avoid to synchronize with predators. The mathematical expressions of an agent updating its direction in systems A and B are

$$\begin{array}{c} e^{i\theta_{A,w}\left(t+1\right)}\\ \;\;\;\;=e^{i\Delta \theta_{A,w}\left(t\right)}\frac{\sum_{j\in \Gamma_{Aw}\left(t+1\right)}e^{i\theta_{A,j}\left(t\right)}+\sum_{j\in \Omega_{AB,w}\left(t+1\right)}e^{i\theta_{B,j}\left(t\right)}}{\|\sum_{j\in \Gamma_{Aw}\left(t+1\right)}e^{i\theta_{A,j}\left(t\right)}+\sum_{j\in \Omega_{AB,w}\left(t+1\right)}e^{i\theta_{B,j}\left(t\right)}\|}, \end{array}$$ (9)

and

$$\begin{array}{c} e^{i\theta_{B,w}\left(t+1\right)}\\ \;\;\;\;=e^{i\Delta \theta_{B,w}\left(t\right)}\frac{\sum_{j\in \Omega_{BA,w}\left(t+1\right)}e^{i\theta_{A,j}\left(t\right)}-\sum_{j\in \Gamma_{Bw}\left(t+1\right)}e^{i\theta_{B,j}\left(t\right)}}{\|\sum_{j\in \Omega_{BA,w}\left(t+1\right)}e^{i\theta_{A,j}\left(t\right)}-\sum_{j\in \Gamma_{Bw}\left(t+1\right)}e^{i\theta_{B,j}\left(t\right)}\|}, \end{array}$$ (10)

respectively. Note that this model can be potentially applied to coupled systems such as a flocking of birds/ducks interacting with a schooling of fish, where the birds/ducks eat the fish.

### (3) Competitive relationship

The competitive relationship between two SPA systems has negative effects on both since the food, space or other resources that they competing for are limited [37]. Thus, the direction of each agent is obtained by the average directions of the neighboring agents of its own subsystem and opposite directions of the neighboring agents in the other subsystem, whose mathematical expression is:

$$e^{i\theta_{A,w}\left(t+1\right)}=e^{i\Delta \theta_{A,w}\left(t\right)}\frac{\sum_{j\in \Gamma_{Aw}\left(t+1\right)}e^{i\theta_{A,j}\left(t\right)}-\sum_{j\in \Omega_{AB,w}\left(t+1\right)}e^{i\theta_{B,j}\left(t\right)}}{\|\sum_{j\in \Gamma_{Aw}\left(t+1\right)}e^{i\theta_{A,j}\left(t\right)}-\sum_{j\in \Omega_{AB,w}\left(t+1\right)}e^{i\theta_{B,j}\left(t\right)}\|}.$$ (11)

According to Eq (11), one can obtain the direction changes of agents in system B symmetrically.

### (4) Network of self-propelled agent systems

**Fig 2:**
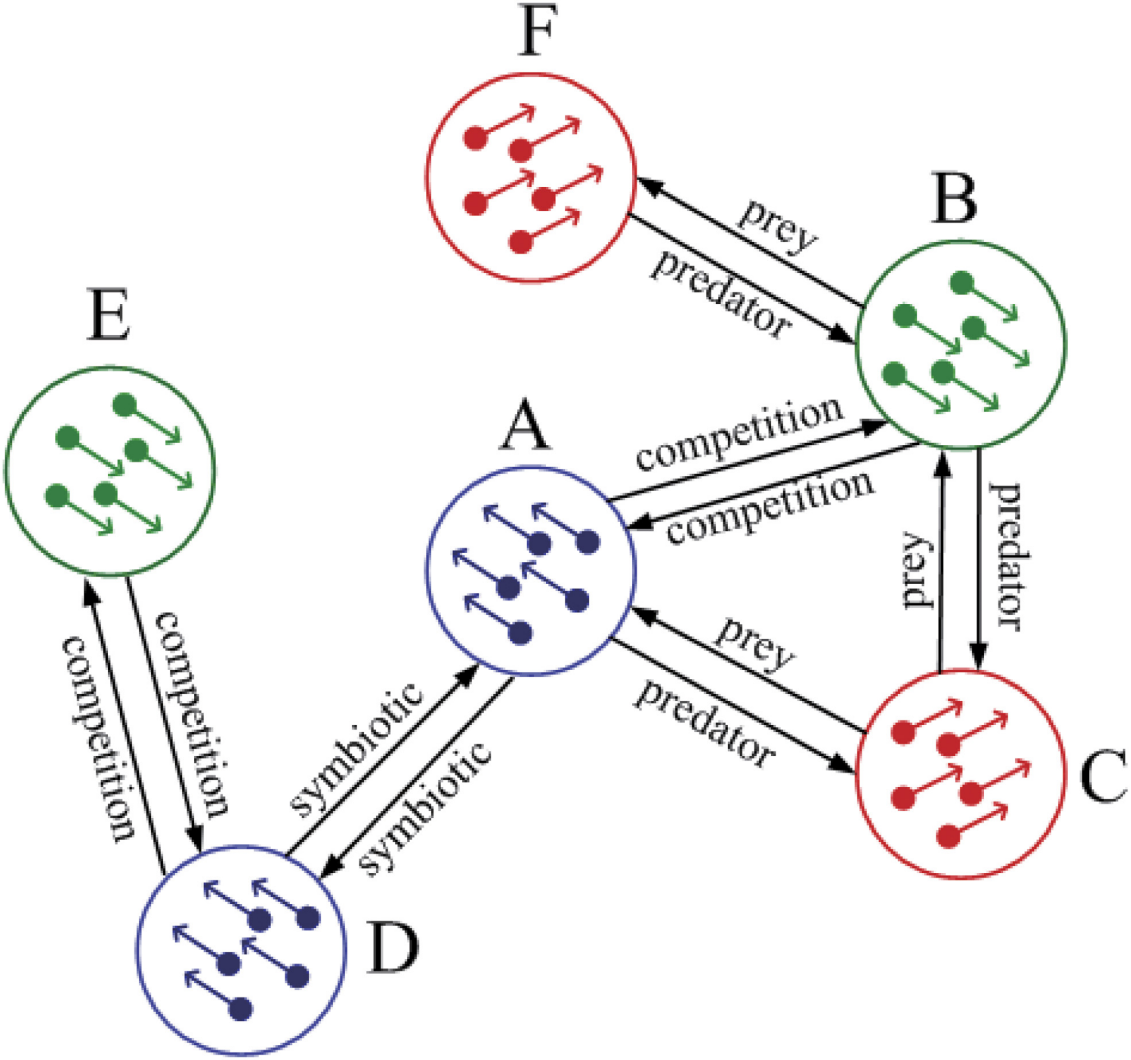
Illustrate a simple network of self-propelled agent systems. This system is composed of 6 SPA subsystems, showing that: (i) if two sub-systems are connected by the symbiotic relationship, the two subsystems tend to synchronize to the same direction (for example A and D); (ii) if two subsystems are connected by the competitive relationship, the two subsystem tend to synchronize with opposite directions (for example A and B); (iii) if two subsystems are connected by the predator-prey relationship, the two subsystem tend to synchronize with different directions (for example A and C).

In real world, usually more than two SPA systems are coupled with each other, which is also addressed in this work. As shown in Fig 2, there are 6 SPA subsystems coupled together by different relationships between each pair of subsystems. In such a network of SPA systems, different subsystem may converge to different level of synchronization, thus we define a vector of order parameter *V*_*s*_ as

$$V_{s}=\left[V_{\alpha },V_{\alpha 1},\ldots ,V_{\alpha k},\ldots ,V_{\alpha N}\right],$$ (12)

where *V*_*α*_ is the synchronization degree for the entire system, which denotes the synchronization of all the agents of *n* subsystems. *V*_*αw*_ (*w* = 1, 2, …, *N*) represents the synchronization of system *w*. The order parameter not only represents the degree of each subsystem, but also contains the synchronization of the entire system.

Considering the situation without noise (i.e. noise amplitude *η* = 0), we simulate the synchronization of networks of SPA systems coupled by three different relationships, and it can help us understand the behavior of a network of *n* SPA systems. Moreover, we also investigate the effect of the four important parameters on the synchronization of a network of SPA systems, namely the coupling strength *β*, the radius *R*, the absolute speed *v*_0_, and the system size *n*. In the simulations, we use the periodic boundary condition [13] and the fixed density [1] and the density of the system is *ρ* = *n*_*k*_/*L* ^2^ = 1. If there is no special statement, we use the parameters as *R* = 0.3, *v*_0_ = 0.1, *n*_*k*_ = *n* = 200 for each subsystem and all the results are averaged over 400 realizations.

## Network of SPA systems coupled by symbiosis relationship

In this section, we show that how these three factors: the system size *n*, the absolute velocity *v*_0_, and the radius *R*, affect the synchronization of two SPA subsystems coupled by symbiotic relationships.

**Fig 3:**
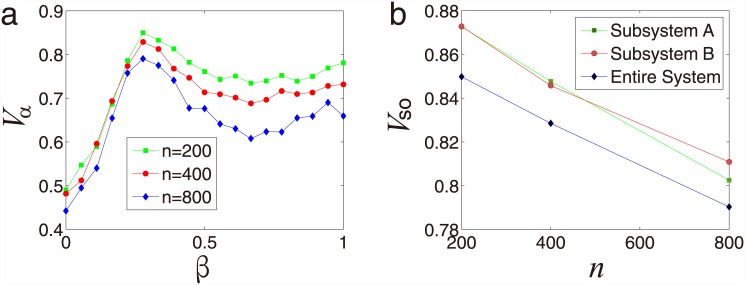
System size effect for SPA systems coupled by symbiotic relationship. (a) Synchronization *V*_*α*_ as a function of the coupling strength *β* under different system size *n* of two subsystems, exhibiting that there exists an optimal value of *β*, when *β* = *β*_*o*_ the the entire system reaches an optimal synchronization. (b) Optimal synchronization *V*_*so*_ as a function of system size *n* for subsystem A, subsystem B and the entire system. All the data points are in S1 File.

In Fig 3(a), the synchronization *V*_*α*_ is a function of the coupling strength *β* under three different values of system size *n*. The results demonstrate that *V*_*α*_ increases and then decreases with coupling strength *β* increasing, indicating that there exists an optimal value of *β*_*o*_ and when *β* = *β*_*o*_ the system achieves optimal synchronization *V*_*so*_. As shown in Fig 3(b), the optimal synchronization decreases with the system size for both the subsystems and the entire system.

**Fig 4:**
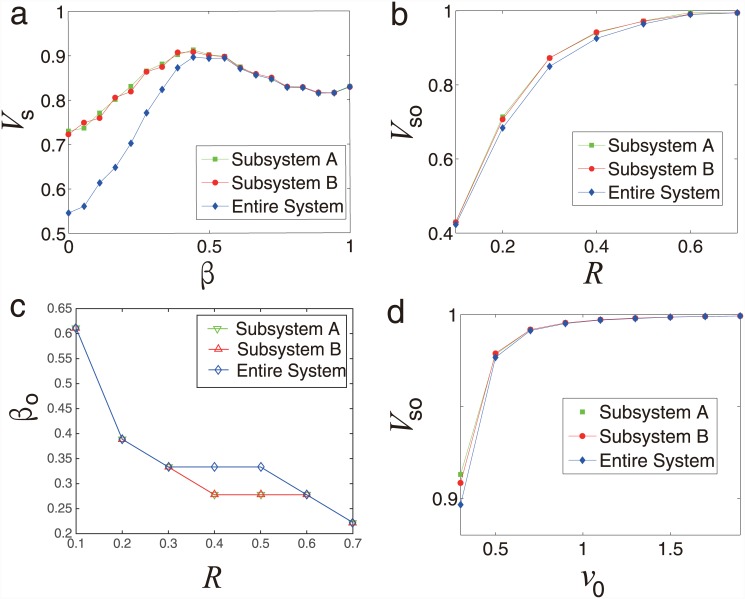
Absolute velocity and radius effect for symbiotic relationship. (a) Synchronization *V*_*s*_ as a function of the coupling strength *β* for subsystem A, subsystem B and the entire system. (b) Optimal synchronization *V*_*so*_ as a function of radius *R* for subsystem A, subsystem B and the entire system. (c) Optimal coupling strength *β*_*o*_ as a function of radius *R* for subsystem A, subsystem B and the entire system. (d) Optimal synchronization *V*_*so*_ as a function of the absolute velocity *v*_0_ for subsystem A, subsystem B and the entire system. The simulation results are saved in S2 and S3 Files.

As shown in Fig 4(a), *V*_*s*_ increases and then decreases showing a peak as the coupling coefficient *β* increasing. As shown in Fig 4(b), the optimal synchronization *V*_*so*_ is an monotonously increasing function of *R* and each subsystem and entire system reach fully synchronized when *R* > 0.7. We also find that the optimal coupling strength *β*_*o*_ decreases as *R* increasing in each subsystem and entire system [Fig 4(c)]. Furthermore, as shown in Fig 4(d), the optimal synchronization *V*_*so*_ increases as *v*_0_ increasing, indicating that the absolute velocity *v*_0_ could increase the synchronization of each subsystem and entire system. In summary, as we know, when *β* = 0, the system is equivalent to two isolated systems with the same density *ρ* = 1; and for the special case that *β* = 1, the network is equivalent to each subsystem with density *ρ* = 2. However, the case *β* = 1 does not correspond to the optimal synchronization. Counter-intuitively, we find that the coupled system with symbiotic relationship shows an optimal value of *β*, indicating that we can design optimal coupled system by choosing suitable value of coupling strength.

## Network of SPA systems coupled by predator-prey relationship

Different SPA systems may coupled by the predator-prey relationships, and its synchronization might be influenced by the properties of each SPA system and their relationships. Next we present how the system size *n*, the absolute velocity *v*_0_ and the radius *R* affect the synchronization of the network of SPA systems coupled by predator-prey relationships.

**Fig 5:**
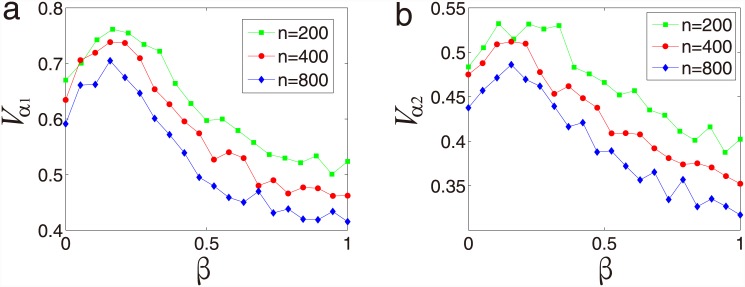
System size effect for predator-prey relationship. (a) Synchronization *V*_*α*1_ as a function of the coupling strength *β* for the subsystem of predators. (b) Synchronization *V*_*α*2_ as a function of the coupling strength *β* for the subsystem of preys. All the data points are in S4 File.

We denote *V*_*α*1_ and *V*_*α*2_ as the synchronization of the subsystem of predators and the subsystem of preys, the behaviors of which under different system size *n* as the coupling strength *α* increasing is showing in Fig 5(a). Both the degree of synchronization of these two subsystems present a peak as the coupling strength *α* increasing, as shown in Fig 5. The peak value *V*_*so*_ decrease as the system size *n* increasing.

**Fig 6:**
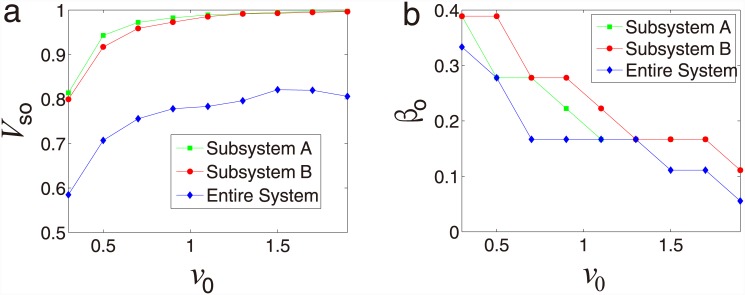
Absolute velocity effect for network of SPA systems coupled by predator-prey relationship. (a) Optimal synchronization *V*_*so*_ as a function of the absolute velocity *v*_*o*_ for each subsystem and the entire system. (b) Optimal coupling strength *β*_*o*_ as a function of the absolute velocity *v*_0_ for each subsystem and the entire system. The simulation results are saved in S5 File.

We next study the effect of absolute velocity on synchronization for the paired SPA system with predator-prey relationships. In Fig 6(a), the optimal synchronization *V*_*so*_ for each subsystem and the entire system varies with the absolute velocity *v*_0_. The results demonstrate that, *V*_*so*_ monotonously increase with the velocity *v*_0_ increasing, which implies that the synchronization is significantly improved when the velocity *v*_0_ increases. In particular, the synchronization converges to full synchronization when velocity *v*_0_ is large. Besides, for a fixed velocity *v*_0_, the synchronization of each subsystem is better than the case of the entire system. To further study the difference between the synchronization of each subsystem and the entire system, we treat the corresponding optimal value *β*_*o*_ as a function of the absolute velocity *v*_0_ [Fig 6(b)] and find that optimal value *β*_*o*_ decreases quickly as the absolute velocity *v*_0_ increases for each subsystem and the entire system. Especially, for a fixed velocity *v*_0_, the optimal value *β*_*o*_ of each subsystem is greater than that of the entire system.

**Fig 7:**
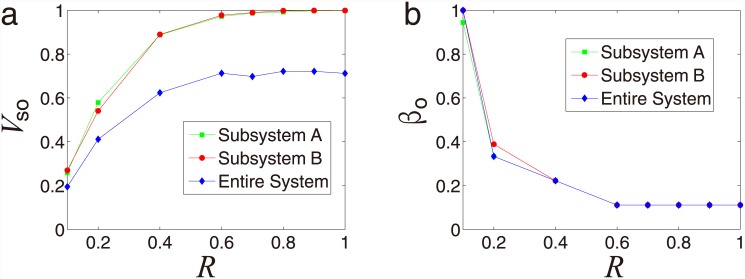
Radius effect for predator-prey relationship. (a) Optimal synchronization *V*_*so*_ as a function of radius *R* for each subsystem and the entire system. (b) Optimal coupling strength *β*_*o*_ as a function of radius *R* for each subsystem and the entire system. All the data points are in S6 File.

In Fig 7(a), the optimal synchronization *V*_*so*_ can be achieved with the radius *R* for each subsystem and the entire system with various radius *β*. For a fixed *β*, *V*_*so*_ is an increasing function of radius *R*, which implies that the synchronization is significantly improved when the radius *R* increases. In particular, when *R* is large enough each subsystem achieve a full synchronization but the entire system goes to a constant level of synchronization (but less than 1), indicating that the predator subsystem and the prey subsystem converges to different directions as illustrated in Fig 1 as well. To further study the difference between the synchronization of each subsystem and the entire system, we regard the corresponding optimal value *β*_*o*_ as a function the radius *R* in Fig 7(b) and find that optimal value *β*_*o*_ decreases as the radius *R* increases for each subsystem and the entire system.

## Network of SPA systems coupled by competitive relationship

**Fig 8:**
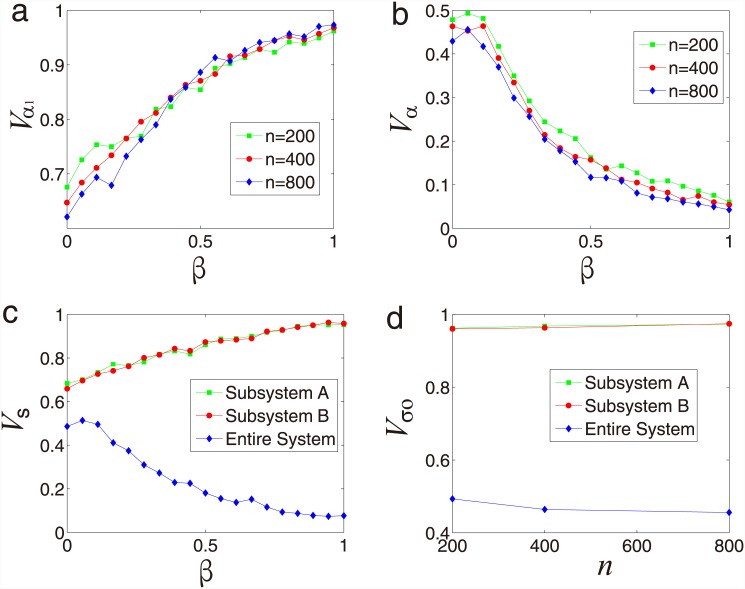
System size and absolute velocity effect for competition relationship. (a) Synchronization *V*_*α*1_ as a function of the coupling strength *β* for each subsystem. (b) Synchronization *V*_*α*_ as a function of the coupling strength *β* for the entire system. (c) Synchronization *V*_*s*_ as a function of the coupling strength *β* for each subsystem and the entire system. (d) Optimal synchronization *V*_*so*_ as a function of system size *n* for each subsystem and the entire system. The simulation results are saved in S7 and S8 Files.

We study the synchronization of the paired SPA systems coupled by competitive under different system size *n*, radius *R*, and different values of velocity *v*. As a function of the coupling strength for different system size *n*, the synchronization of subsystem A *V*_*α*1_ monotonically increases as the coupling strength *β* increases, as shown in Fig 8(a). On the contrary, when we consider synchronization of the entire system *V*_*α*_ as a function of the coupling strength *β*, we can see that *V*_*α*_ shows a peak at small *β*, indicating that tiny competition can increase the synchronization of the entire system. This result is as surprising as the result presented in Fig 8(b) that small noise can improve the synchronization of a system.

Besides, we show the synchronization *V*_*s*_ as a function of the absolute velocity *β* for each subsystem and the entire system [Fig 8(c)]. Our simulation results indicate that, the difference between each subsystem and the entire system synchronization changes sharply as the coupling strength *β* increases. In particular, the total synchronization of the entire system is significantly less than each subsystem when the coupling strength *β* increases. The simulation results demonstrate *V*_*s*_ of each subsystem is better than the case of the entire system, since the synchronization of one system benefits the synchronization of another system, but the synchronized direction of each subsystem is different from each other. In order to show the optimal synchronization *V*_*so*_ as a function of system size *n*, we perform the the numerical simulations in Fig 8(d). Our simulation results suggest that, for each subsystem and the entire system, the optimal synchronization *V*_*so*_ maintain a constant value as the system size *n* increases.

## Conclusion

SPA systems usually interact or depend on each other, forming networks of SPA systems. In this work, we propose a model to mimic the synchronization of the network of SPA systems coupled by different relationships: symbiosis, predator-prey, and competition. The synchronization process in the networks of SPA systems could be influenced by both the properties of single SPA systems (the size of each subsystem *n* and the absolute velocity) and the relationship between different SPA systems (the coupling strength *β* and the radius *R*). Moreover, the networks coupled SPA systems coupled by different relationships show different behaviors: (1) the system coupled with symbiotic relationships shows a complete synchronization, as each subsystem reaching a complete synchronization, under high absolute velocity or high radius; (2) the system coupled by the predator-prey relationships shows an optimal but not complete synchronization, while each subsystem can arrive at a complete synchronization; (3) the system of coupled competitive SPA systems shows an optimal synchronization for small coupling strength between SPA systems, while no optimal synchronization for each subsystems. These interesting results can significantly improve our understanding of the synchronization principles of complex systems.

## Supporting Information

S1 File S1.mat (Fig 3). The simulation results about two subsystems with symbiosis relationship, when *n* various.
(MAT)

S2 File S2.mat (Fig 4). The simulation results about two subsystems with symbiosis relationship, when *v*_0_ various.
(MAT)

S3 File S3.mat (Fig 4). The simulation results about two subsystems with symbiosis relationship, when *R* various.
(MAT)

S4 File S4.mat (Fig 5). The simulation results about two subsystems with predator-prey relationship, when *n* various.
(MAT)

S5 File S5.mat (Fig 6). The simulation results about two subsystems with predator-prey relationship, when *v*_0_ various.
(MAT)

S6 File S6.mat (Fig 7). The simulation results about two subsystems with predator-prey relationship, when *R* various.
(MAT)

S7 File S7.mat (Fig 8). The simulation results about two subsystems with competition relationship, when *n* various.
(MAT)

S8 File S8.mat (Fig 8). The simulation results about two subsystems with competition relationship, when *v*_0_ various.
(MAT)
